# Supplementary material for: End Sequence Analysis Toolkit (ESAT) expands the extractable information from single-cell RNA-seq data
Source: Genome Res. 2016 Oct;26(10):1397–410. doi: 10.1101/gr.207902.116 (PMC5052061; doi:10.1101/gr.207902.116)
Supplement: Supplemental Material [file supp_gr.207902.116_Supp_Materials.tar.gz › ESAT-esat_v0.1/doc/html/classbroad_1_1pda_1_1enrichment_1_1_enrichment_metric-members.html]

ESAT: Member List


ESAT


- Main Page
- Related Pages
- Packages
- Classes
- Files

- Class List
- Class Index
- Class Hierarchy
- Class Members

All Classes Namespaces Files Functions Variables Enumerator Pages

- broad
- pda
- enrichment
- EnrichmentMetric

broad.pda.enrichment.EnrichmentMetric Member List

This is the complete list of members for broad.pda.enrichment.EnrichmentMetric, including all inherited members.

|  |  |  |
| --- | --- | --- |
| EnrichmentMetric(String name, String dataType1, String dataType2, String statisticName, String method, double statistic, double pvalue, int totalCount, int regionCount) | broad.pda.enrichment.EnrichmentMetric |  |
| getDataType1() | broad.pda.enrichment.EnrichmentMetric |  |
| getDataType2() | broad.pda.enrichment.EnrichmentMetric |  |
| getDataTypes() | broad.pda.enrichment.EnrichmentMetric |  |
| getId() | broad.pda.enrichment.EnrichmentMetric |  |
| getMethod() | broad.pda.enrichment.EnrichmentMetric |  |
| getPValue() | broad.pda.enrichment.EnrichmentMetric |  |
| getStatistic() | broad.pda.enrichment.EnrichmentMetric |  |
| getSubmethod() | broad.pda.enrichment.EnrichmentMetric |  |
| name | broad.pda.enrichment.EnrichmentMetric | protected |
| statistic | broad.pda.enrichment.EnrichmentMetric | protected |
| statisticName | broad.pda.enrichment.EnrichmentMetric | protected |
| toString() | broad.pda.enrichment.EnrichmentMetric |  |
| totalCount | broad.pda.enrichment.EnrichmentMetric | protected |


---

Generated on Fri Aug 15 2014 15:10:59 for ESAT by  

 1.8.7
